# A PFM-Based Calibration Method for Low-Power High-Linearity Digital Pixel

**DOI:** 10.3390/s25010252

**Published:** 2025-01-04

**Authors:** Yu Cheng, Jionghan Liu, Xiyuan Wang, Hongyu Hou, Qian Jiang, Yuchun Chang

**Affiliations:** School of Integrated Circuits, Dalian University of Technology, Dalian 116000, China; cyuhope@mail.dlut.edu.cn (Y.C.);

**Keywords:** PFM, digital pixel, DROIC, PbSe

## Abstract

The nonlinearity problem of digital pixels restricts the reduction in power consumption at the pixel-level circuit. The main cause of nonlinearity is discussed in this article and low power consumption is attained by reducing the static current in capacitive transimpedance amplifiers (CTIAs) and comparators. Linearity was successfully improved through the use of an off-chip calibration method. A 64 × 64 array prototype digital readout integrated circuit (DROIC) was fabricated using a 0.18 μm 1P6M CMOS process. Experimental results indicated that the post-calibration linearity reached 99.6% with an input current of up to 1.5 μA. The static power consumption per digital pixel was 6 μW.

## 1. Introduction

In recent years, infrared detection technology has been increasingly utilized in various applications, such as security systems, driving assistance systems, and military equipment [1,2,3,4]. Among various infrared detector materials, PbSe has gained attention due to its unique properties. As a mid-wave infrared (MWIR) detector material [5], PbSe responds to the 3–5 µm spectral range, and by adjusting the material composition, even broader spectral response ranges can be achieved. At room temperature (300 K) [6], PbSe exhibits high detectivity and a response speed on the microsecond timescale. This makes PbSe suitable for detecting fast-moving objects, such as in autonomous driving and missile detection applications [7,8]. Since it operates at room temperature without the need for cooling devices, it significantly reduces the size and power consumption of detection systems. Another characteristic of PbSe is its higher output current range compared to other materials [9,10], requiring the readout circuit to have greater charge handling capability.

For conventional readout integrated circuits (ROICs), pixel output analog signals are converted to digital through column-level analog-to-digital converters (ADCs) [11]. The charge handling capability is limited by pixel area and swing, while the frame rate is constrained by ADC speed and array size, making it difficult to meet the high-speed, high-current characteristics of PbSe. Due to their advantages of high dynamic range and high frame rate, digital pixels have been widely reported [12,13,14]. Generally, there are two methods of achieving digital outputs: in-pixel ADC and pulse frequency modulation (PFM). The former integrates ADCs at the pixel level, which increases the frame rate but does not improve the charge handling capacity. The latter generates reset signals internally to clear charges on integration capacitors, thereby enhancing charge handling capability. PFM-based designs have demonstrated a superior signal-to-noise ratio (SNR) and dynamic range performance [6,8].

PbSe is a photoconductive detector, and a capacitive transimpedance amplifier (CTIA) is used as the input stage for PFM-based digital pixels [15], due to its ability to provide a more stable bias and higher injection efficiency. A comparator is used to compare the integrated voltage and generate a reset signal. The frequency of the reset signal is proportional to the input signal current [16]. Dealing with higher input currents requires operational amplifiers and comparators with greater bandwidth. However, this leads to an increase in power consumption. Insufficient bandwidth can lead to nonlinear output [17]. To address this, a soft reset mechanism has been introduced [18]. The soft reset mechanism involves the use of non-overlapping phases and an additional capacitor, which inevitably increases area and power consumption [15].

In this study, an analysis of the nonlinearity of PFM digital pixels was conducted, and an off-chip calibration method was proposed to mitigate these nonlinearities. Analysis revealed a linear relationship between the difference in reset voltages and flip voltages and the input current. Taking advantage of this property, an off-chip calibration was implemented in this paper. A prototype in the form of a 64 × 64 DROIC array was fabricated to validate the calibration method. The proposed method offers the benefit of reduced power consumption and improved linearity, and is particularly applicable to detectors that generate large currents.

## 2. PFM-Based Digital Pixel and Nonlinearity

**Figure 1:**
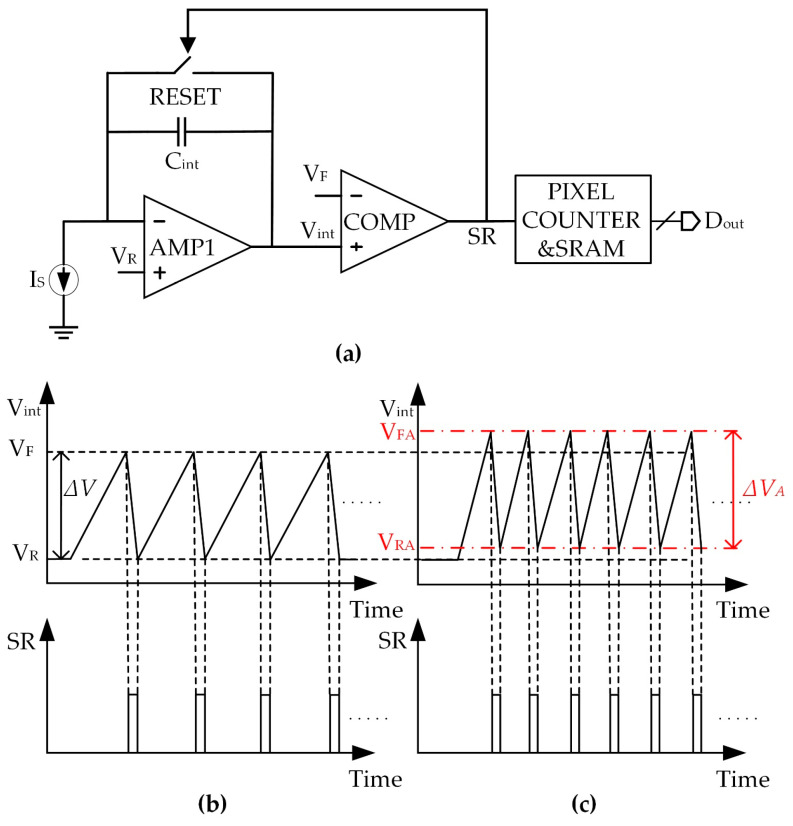
(**a**) Conventional PFM-based digital pixel structure; (**b**) ideal signal of the digital pixel; (**c**) actual signal with large current as input.

A schematic of a conventional PFM-based digital pixel is shown in Figure 1a. In the operation of the digital pixel, the CTIA output is initially set to the reference voltage ($V_{R}$) during the reset phase. As the exposure phase begins, the current $I_{s}$ is integrated into the capacitor ($C_{int}$). When the voltage $V_{int}$ reaches the flip voltage $V_{F}$, the comparator generates the self-reset (SR) signal for the CTIA and also serves as an input for the counter. Ideally, the SR signal resembles a series of pulses with a frequency directly related to the magnitude of $I_{s}$ [19], as shown in Figure 1b. The counter will accumulate the SR signals during the exposure time $T_{int}$ and store the data in SRAM for subsequent readout. Under ideal conditions, the digital output can be expressed as

(1) $$N=\frac{T_{int}\cdot I_{s}}{∆V\cdot C_{int}}$$

where V means the difference between $V_{F}$ and $V_{R}$. In an ideal case, $∆V=V_{F}-V_{R}={∆V}_{S}$, and there is a linear relationship between the input current and the output count.

In practice, it is a challenge to implement a comparator without delay, which means the comparator may not flip precisely when $V_{int}$ reaches $V_{F}$, as shown in Figure 1c. $V_{int}$ will continue to integrate and exceed $V_{F}$ to a new level (denoted as $V_{FA}$), at which point the SR pulse will be generated. The over-integrated voltage will increase with the input current increasing. Once the self-reset signal is triggered, $V_{int}$ will rapidly decrease. The short reset time for the CTIA necessitates that AMP1 has a wide gain–bandwidth product (GBW) to ensure that $V_{int}$ can be discharged precisely to the baseline voltage $V_{R}$. If the GBW of AMP1 is limited, the reset voltage may not return exactly to $V_{R}$, leading to a new voltage difference, which is referred to as ${∆V}_{A}$ in this paper.

Based on the analysis, the over-integrated voltage $V_{FA}$ can be written as

(2) $$V_{FA}=V_{F}+\frac{I_{S}\times t_{p}}{C_{int}}$$

where $t_{p}$ is the delay of comparator, as expressed in [20].

(3) $$t_{p}=\tau_{C}\times \ln\frac{2k}{2k-1}$$

(4) $$k=\frac{∆V}{V_{min,comp}}$$

(5) $$V_{min,comp}=\frac{V_{OH}-V_{OL}}{A_{VC}}$$

where $\tau_{C}=1/\omega_{C}$, $\omega_{C}$ is −3 dB bandwidth, $V_{OH}$ and $V_{OL}$ represent the output voltage of high level and low level, and $A_{VC}$ is the open-loop gain. By utilizing Taylor series expansion and neglecting higher-order terms, Equation (3) can be rewritten as

(6) $$t_{p}=\tau_{C}\times \left(\frac{2k}{2k-1}-1\right)=\frac{\tau_{C}}{2k-1}.$$

Once the self-reset signal is generated, the system goes into discharge state. The discharge equation is given by

(7) $$V_{int}\left(t\right)=V_{FA}\times e^{-\frac{t}{\tau_{A}}}$$

where $\tau_{A}=1/\omega_{A}$, and $\omega_{A}$ is the unity gain bandwidth of AMP1. We assume the discharge time is $t_{H}$; then, $V_{RA}$ is found as

(8) $$V_{RA}=V_{FA}\times e^{-\frac{t_{H}}{\tau_{A}}}.$$

Thus,

(9) $${{{∆V}_{A}=V}_{FA}-V}_{RA}=V_{FA}\times \left(1-e^{-\frac{t_{H}}{\tau_{A}}}\right).$$

Substituting Equation (2) into Equation (9),

(10) $${∆V}_{A}=V_{F}\times \left(1-e^{-\frac{t_{H}}{\tau_{A}}}\right)+\frac{\tau_{C}}{C_{int}\times \left(2k-1\right)}\times \left(1-e^{-\frac{t_{H}}{\tau_{A}}}\right)\times I_{S}.$$

**Figure 2:**
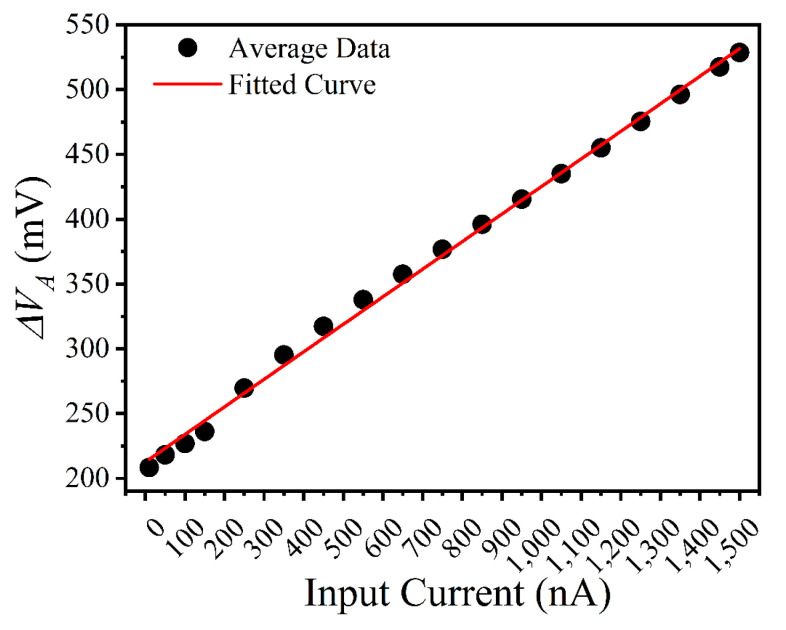
Simulation data of ${∆V}_{A}$ versus input current.

The formula shows that ${∆V}_{A}$ is related to the comparator speed, operational amplifier bandwidth, and input current. There is a linear relationship between ${∆V}_{A}$ and the input current, and when the comparator and operational amplifier are faster, the slope of this linear relationship becomes smaller. It can be observed that the amount of charge corresponding to each integration reset process increases as the integration current increases. When power consumption is limited or the input current is large, if ∆V  in Equation (1) is not corrected, the resulting count value will deviate from the actual value. And as the input current increases, the output nonlinearity becomes more evident. This is the main cause of nonlinearity when dealing with large input currents. Schematic-level simulation is performed and linear fitting is applied to the ${∆V}_{A}$ data. Simulation data also confirm the linear relationship between ${∆V}_{A}$ and $I_{S}$, as shown in Figure 2.

## 3. DROIC Design

**Figure 3:**
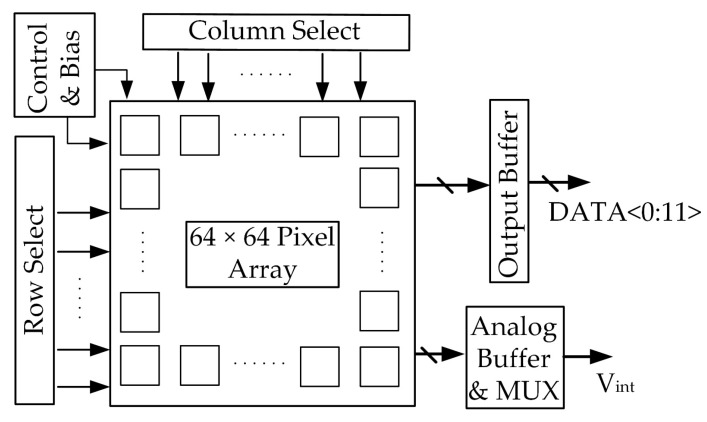
Block diagram of the prototype.

The prototype DROIC is fabricated using a 0.18 μm 1-poly 6-metal CMOS process. The block diagram of DROIC is shown in Figure 3. The core of the DROIC consists of a 64 × 64 array of PFM-based digital pixels, column and row selectors, and an output buffer. Additionally, an Analog Buffer & MUX module is used to acquire the data for calibration.

**Figure 4:**
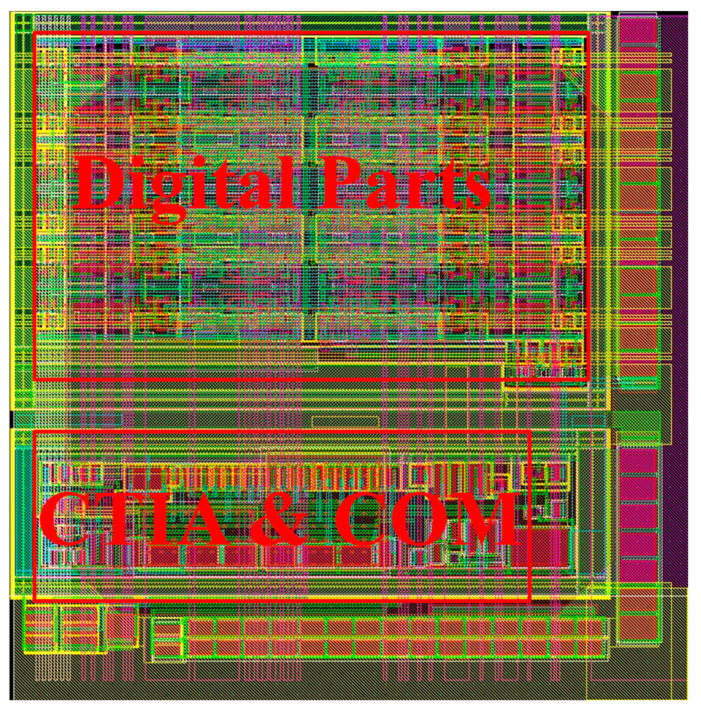
Layout of the digital pixel.

The digital pixels are based on Figure 1. A current source is used to imitate the detector. The current level is set to be adjustable based on the PbSe detector. The telescopic cascode structure is chosen for the AMP1 and comparator because it can achieve high gain and wide GBW at low currents. The tail current for the AMP1 and comparator is 1.8 μA and 1.5 μA, respectively. Under a power supply of 1.8 V, the static power consumption of the digital pixel is approximately 6 μW. The pixel layout is shown in Figure 4. The pixel size is 50 μm × 50 μm, with a 50 fF integrated capacitor, a 12-bit counter, and SRAM. Most of the pixel size is occupied by digital circuits.

To obtain the data for the calibration method, the $V_{int}$ signal of some pixels can be output externally through the Analog Buffer & MUX module. A total of 10 pixels of $V_{int}$ in the pixel array are connected to this module. One of the $V_{int}$ outputs is selected by MUX and sent outside the chip to measure ${∆V}_{A}$.

**Figure 5:**
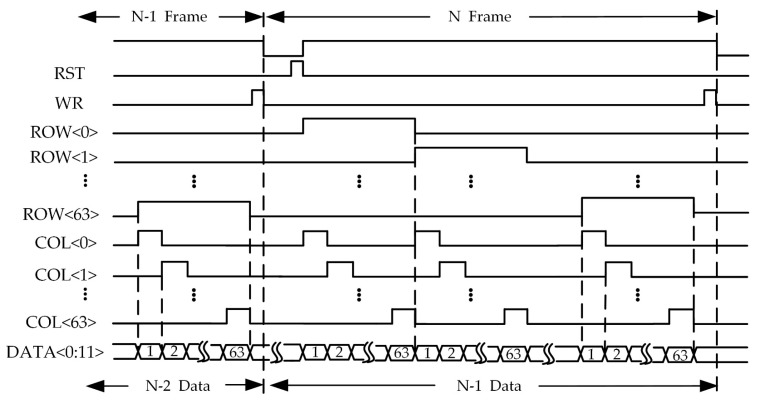
Timing diagram of the prototype.

To achieve a higher frame rate, the prototype can operate in integration-while-read (IWR) mode, with the timing diagram shown in Figure 5. Before integration begins, the RST signal resets the circuit to its initial state. When the INT signal arrives, integration and counting start. At the end of integration, the WR signal writes the counter value to the SARM. When the integration signal for the next frame begins, the data from the previous frame are transferred to the output buffer and read out by controlling the ROW and COL signals. The readout time of the data is shorter than the integration time to ensure that the data from the previous frame are fully read out before the next WR signal arrives.

## 4. Measurement Results

**Figure 6:**
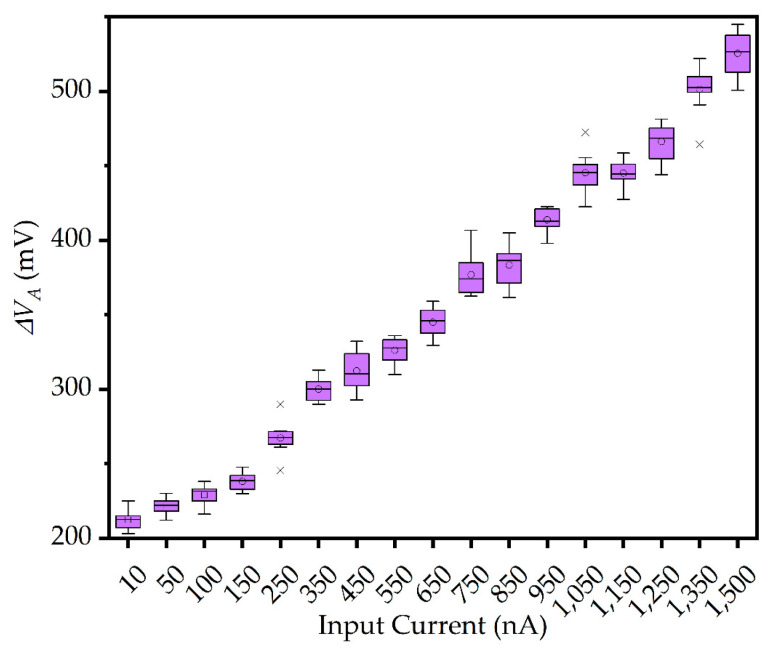
$∆V_{A}$ of the pixel versus input current.

$V_{R}$ and $V_{F}$ were generated by the bias circuit, with values of 0.8 V and 1 V, respectively. Different input currents from 10 nA to 1500 nA were applied to the pixels and the resulting $\;{∆V}_{A}$ was measured as shown in Figure 6. A similar linear relationship exists between the measured data and the simulation results, which indicates a consistent behavior of the system.

**Figure 7:**
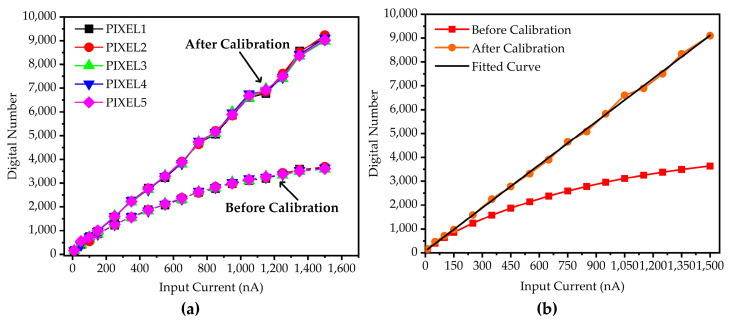
Relation between the digital output and input current: (**a**) data of five pixels; (**b**) average value of the array.

The average value of the data was used to calibrate the obtained digital code, as shown in Figure 6, and the calibration formula is as follows:

(11) $$N_{c}=N_{A}\times \frac{\bar{∆V_{A}}}{∆V_{S}}$$

where $\bar{∆V_{A}}$ is the average value of the measured data. $N_{A}$ is the digital output of the pixel array. The data before and after calibration are shown in Figure 7.

Five pixels inside the array were selected for calibration, which were located at the center and four corners of the array. Figure 7a demonstrates that the nonlinear trends were successfully calibrated. The differences between the five calibrated pixels may be attributed to the mismatch in the pixel array and in the current mirror used to provide the input current. In Figure 7b, we use the average value of 64 × 64 array before and after calibration. The coefficient of determination of the linear fitted curve for the post-calibration data is 0.9991, indicating a high correlation between the average data points and the fitted curve. This value suggests that the calibration process was highly effective in reducing nonlinearity and improving the accuracy of the system response.

**Figure 8:**
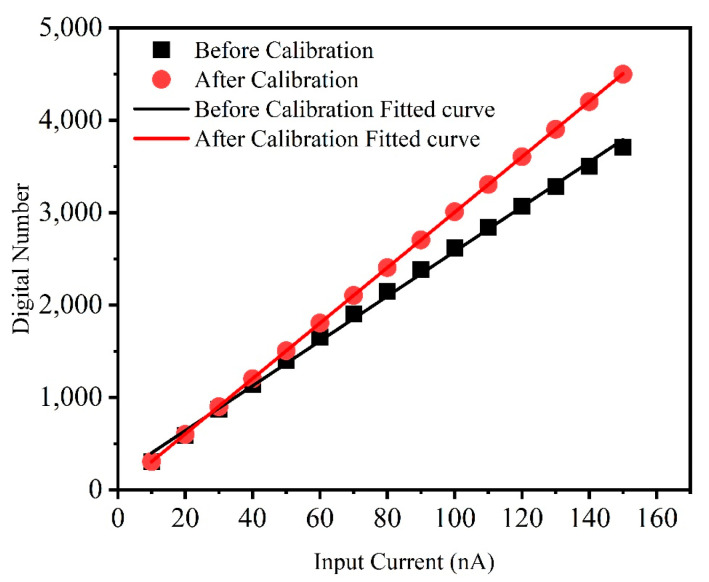
Relation between the digital output and input current with long integration time.

When the input current is low or the integration time is short, meaning the total input charge is small, the count value will be relatively low, leading to less significant nonlinearity. As shown in Figure 7, the difference in digital number before and after calibration is small. To demonstrate the applicability of the calibration method proposed in this paper, a set of data with a long integration time was measured, where the integration time was 1 ms. Linear fitting was applied to the data as shown in Figure 8. The data represent the average value of 4096 pixels. The coefficient of determination of the fitted curve before and after calibration is 0.9979 and 0.9999, respectively. Since the deviation of $∆V_{A}$ is smaller at low input current than at high input current, the nonlinearity is less significant at lower currents. However, the calibration method proposed in this paper remains effective.

Table 1 shows the performance comparison of this work and some reported DROICs for IRFPAs. For the PbSe photoconductive detector, a CTIA is typically used as the input stage. Although it consumes more power than a DI, it can provide a more stable voltage bias. This work uses the most classic PFM structure, and through the correction methods, it maintains linearity in the output at a maximum input current of 1.5 μA, thereby reducing the power consumption of the readout circuit at high current inputs. A soft reset circuit was added within the pixel in [15], achieving good linearity under large current conditions. However, its static power consumption was about 1.6 times that of the proposed work. Additionally, the integration capacitor used was 200 fF, and a larger integration capacitor reduces the auto-reset frequency, resulting in lower power consumption compared to the 50 fF integration capacitor used in this work. The calibration method proposed in this paper demonstrates a more significant reduction in power consumption. When the pixel operates in IWR mode, the chip’s frame rate can reach 4000 fps (frames per second), better addressing the response speed of PbSe.

## 5. Conclusions

A 64 × 64 array prototype fabricated in a 180 nm CMOS process shows the practical implementation of the calibration method. The effectiveness of the proposed technique is demonstrated by measuring $∆V_{A}$ and successfully calibrating the nonlinearity outside the chip. The nonlinear calibration method introduced for the conventional PFM structure represents a significant advancement in pixel array design. This approach reduces power consumption and improves linearity without the need for assisting circuits within each pixel. Leveraging the simple PFM structure, the prototype achieves a high input range, high linearity, and low static power consumption of 6 μW per pixel.

## Figures and Tables

**Table 1:** Comparison of DROIC performance.

	[15]	[21]	[22]	This Work
Array Size	128 × 128	640 × 512	640 × 512	64 × 64
Pixel Size (μm)	50 × 50	25 × 25	15 × 15	50 × 50
Input Structure	CTIA	CTIA	DI	CTIA
Pixel Type	Digital	Digital	Analog	Digital
Max Input Current	2 μA	60 nA	-	1.5 μA
Resolution (bits)	10	13	-	12
Capacity (Me^−^)	-	350	4.5	560
Frame Rate (fps)	4000	730	300	4000
Static Power	10 μW/pixel	-	100 mW	6 μW/pixel

## Data Availability

The original contributions presented in the study are included in the article; further inquiries can be directed to the corresponding author.

## References

[1] Lee H.-J., Eom J.H., Kang K.-K., Ryu S.-M., Jang A., Kim J.G., Jang H., Kim J., Choi H., Jung H., Ting D.Z., Fulop G.F., Zheng L.L. (2023). 640 × 512 dual-band midwave and longwave infrared focal plane array at i3system. Infrared Technology and Applications XLIX.

[2] Kelleci B., Kisa M.E., Ergen E., Oktay Z.N., Akbulut M., Nuzumlali Ö.L., Ting D.Z., Fulop G.F., Zheng L.L. (2023). Development of 1280 × 1024 15μm pixel pitch ROIC for MWIR and LWIR IIR detectors. Infrared Technology and Applications XLIX.

[3] Zeng Y., Yang S., Liu Y., Bao R., Zhu Z., Lin J., Zhou X., Chen Y., Yin J., Mak P.-I. (2023). A Digital Readout Integrated Circuit Based on Pixel-Level ADC Incorporating On-Chip Image Algorithm Calibration for IRFPA. IEEE Sens. J..

[4] Kim H.-J. (2024). Design of A prototype 128 × 128 ROIC array for 2.6 μm-wavelength SWIR image sensor applications. Integration.

[5] Qiu J., Liu Y., Zhang G., Shi K., Li Y., Luo Y. (2021). Modified vapor phase deposition technology for high-performance uncooled MIR PbSe detectors. RSC Adv..

[6] Mirzaei M.R., Shi Z. (2024). Room-temperature nanostructured PbSe/CdSe mid-infrared photodetector: Annealing effects. J. Vac. Sci. Technol. B.

[7] Kim C.Y., Woo D.H., Lee H.C. (2019). High-Dynamic-Range ROIC With Asynchronous Self-Controlled Two-Gain Modes for MWIR Focal Plane Arrays. IEEE Sens. J..

[8] Chu P., Chen H., Ding R. (2020). A novel linear-logarithmic readout integrated circuit with high dynamic range. Infrared Phys. Technol..

[9] Margarit J.M., Teres L., Serra-Graells F. (2009). A Sub-μW Fully Tunable CMOS DPS for Uncooled Infrared Fast Imaging. IEEE Trans. Circuits Syst. I.

[10] Mirzaei M.R., Shi Z., Mirzaei M.R., Shi Z. (2024). High-performance uncooled PbSe/CdSe nanostructured mid-infrared photodetector with tunable cutoff wavelength. Appl. Phys. Lett..

[11] Ma H., Kong H., Chang C. (2022). Design of a multi-mode digital pixel with conversion data protection. IET Circuits Devices Syst..

[12] Kayahan H., Yazici M., Ceylan Ö., Gurbuz Y. (2014). A new digital readout integrated circuit (DROIC) with pixel parallel A/D conversion and reduced quantization noise. Infrared Phys. Technol..

[13] Chen Y., Yuan F., Khan G. A new wide dynamic range CMOS pulse-frequency-modulation digital image sensor with in-pixel variable reference voltage. Proceedings of the 2008 51st IEEE International Midwest Symposium on Circuits and Systems.

[14] Abbasi S., Galioglu A., Shafique A., Ceylan O., Yazici M., Gurbuz Y. (2017). A PFM-Based Digital Pixel With an Off-Pixel Residue Measurement for Small Pitch FPAs. IEEE Trans. Circuits Syst. II Express Briefs.

[15] Figueras R., Margarit J.M., Vergara G., Villamayor V., Gutierrez-Alvarez R., Fernandez-Montojo C., Teres L., Serra-Graells F. A 128× 128-pix 4-kfps 14-bit Digital-Pixel PbSe-CMOS Uncooled MWIR Imager. Proceedings of the 2018 IEEE International Symposium on Circuits and Systems (ISCAS).

[16] Goto M., Honda Y., Watabe T., Hagiwara K., Nanba M., Iguchi Y., Saraya T., Kobayashi M., Higurashi E., Toshiyoshi H. (2019). Quarter Video Graphics Array Digital Pixel Image Sensing With a Linear and Wide- Dynamic-Range Response by Using Pixel-Wise 3-D Integration. IEEE Trans. Electron Devices.

[17] Abbasi S., Ceylan O., Gurbuz Y. (2019). A DROIC Based on PFM ADCs Employing Over-Integration for Error Shaping. IEEE Trans. Circuits Syst. I Regul. Pap..

[18] Dei M., Figueras R., Margarit J.M., Teres L., Serra-Graells F. Highly linear integrate-and-fire modulators with soft reset for low-power high-speed imagers. Proceedings of the 2017 IEEE International Symposium on Circuits and Systems (ISCAS).

[19] Chen D.G., Matolin D., Bermak A., Posch C. (2011). Pulse-Modulation Imaging—Review and Performance Analysis. IEEE Trans. Biomed. Circuits Syst..

[20] Allen P.E., Holberg D.R. (2012). CMOS Analog Circuit Design.

[21] Jo Y.M., Woo D.H., Kang S.G., Lee H.C. (2016). Very Wide Dynamic Range ROIC With Pixel-Level ADC for SWIR FPAs. IEEE Sens. J..

[22] Altun O., Kepenek R., Tasdemir F., Akyurek F., Tunca C., Akbulut M., Nuzumlali O.L., Inceturkmen E., Andresen B.F., Fulop G.F., Hanson C.M., Miller J.L., Norton P.R. Development of a fully programmable ROIC with 15 μm pixel pitch for MWIR applications. Proceedings of the SPIE Defense + Security.

